# NIMH perspectives on future directions in neuroimaging for mental health

**DOI:** 10.1038/s41386-024-01900-8

**Published:** 2024-06-19

**Authors:** S. Andrea Wijtenburg, Laura M. Rowland, Aleksandra Vicentic, Andrew F. Rossi, Linda S. Brady, Joshua A. Gordon, Sarah H. Lisanby

**Affiliations:** National Institute of Mental Health, National Institutes of Health, Rockville, MD USA

**Keywords:** Medical research, Neuroscience

## Abstract

NIMH’s mission is to transform the understanding and treatment of mental illnesses through basic and clinical research, paving the way for prevention, recovery, and cure. New imaging techniques hold great promise for improving our understanding of the pathophysiology of mental illnesses, stratifying patients for treatment selection, and developing a personalized medicine approach. Here, we highlight emerging and promising new technologies that are likely to be vital in helping NIMH accomplish its mission, the potential for utilizing multimodal approaches to study mental illness, and considerations for data analytics and data sharing.

## Introduction

The mission of the National Institute of Mental Health (NIMH) is to transform the understanding and treatment of mental illnesses through basic and clinical research, paving the way for prevention, recovery, and cure. Neuroimaging technologies have the potential to be pivotal in improving our understanding of mental illness pathophysiology, identifying brain targets for interventions, and predicting of treatment response, all with the goal of developing a personalized medicine approach. Research studies that employ novel neuroimaging technologies are part of NIMH’s basic and translational research agenda and priorities (https://www.nimh.nih.gov/about/strategic-planning-reports, https://www.nimh.nih.gov/funding/opportunities-announcements). This paper highlights emerging positron emission tomography (PET) and magnetic resonance (MR) technologies as well as advances in imaging approaches in model systems in studies of mental health and illness. These technologies have great potential for advancing the field via investigating neural and molecular systems in vivo and for accelerating the discovery of biomarkers for mental illness risk, trajectories, and treatments aligned with NIMH’s mission.

## PET imaging

Recent advances in PET hardware, data analytics, and tracer development have expanded the range of relevant biological targets, enabling focused assessment of neuroinflammation, synaptic function, endocannabinoid system, mitochondrial function, and dopaminergic function. Evidence suggests that neuroinflammation, the activation of glial cells that maintain homeostasis and are the first line of defense against infection and damage [1], may play a critical role in the pathophysiology of different mental disorders [2]. While previous findings with the 18 kDa translocator protein (TSPO) lacked specificity to microglial cells, which are specifically implicated in neuroinflammation [2], new PET tracers, such as [^11^C] CPPC that targets the colony stimulating factor 1 receptor (CSF1R) have been developed to specifically target microglia and thereby neuroinflammation. PET tracers targeting the synaptic vesicle glycoprotein 2 A (SV2A), a transmembrane protein of synaptic vesicles present in all synaptic terminals, has enabled assessments of synaptic density [3]. Changes in PET measures of synaptic density are thought to reflect changes in number of dendritic spines and thus a change in synapse health [3]. The PET tracer, ^11^C-UCB-J, holds promise to provide a better understanding of synaptic dysfunction in mental illnesses and neurodegenerative disorders [4]. Novel PET tracers that probe the endocannabinoid system, including CB1 receptor and FAAH tracers, have considerable promise, given the evidence for involvement of this system in emotion, stress, and cognition [5]. Further, utilizing [^18^F]BCPP-EF, a PET tracer that binds to mitochondrial electron transport chain complex I, has great potential to improve our understanding of mitochondrial dysfunction [6] while newer dopaminergic PET tracers, such as [^11^C]FLB457, are likely to improve our understanding of dopamine release in the prefrontal cortex [7]. Future PET studies with these novel tracers could identify targets for pharmacological interventions for mental disorders.

## Ultra-high field MR

Standard magnetic resonance imaging involves field strengths of 3 Tesla or less and has limited resolution, hampering the ability to resolve fine details in structure or function. Ultra-high field (>7 Tesla) MR imaging provides unprecedented anatomical precision at the submillimeter level, yielding visualization of cortical lamina and columns, nuclei, and hippocampal subfields among other small regions [8]. The increased signal-to-noise ratio (SNR) at an ultra-high field MR strength provides increased sensitivity and specificity of signals that are not detectable, or very difficult to detect compared to the lower field strengths, such as hemodynamic signals from the cortical microvasculature, and signals from chemicals with low concentration involved in neurotransmission, metabolism, and other cellular processes [9]. For example, detection of metabolites that are highly relevant for psychiatric disorders, such as γ-aminobutyric acid, glutamate, and glutamine (Gln), is feasible at 7 T without the use of specialized pulse sequences [10]. In combination with connectivity analyses, ultra-high field MR imaging could provide a better understanding of the distinct functional and structural properties of circuits and networks that support cognitive, social, and emotional processing and how these are altered in clinical populations. Further, application of ultra-high field MR to study development has significant potential to improve our ability to identify both structural and functional developmental abnormalities [11, 12].

## Novel MR methods

Beyond increased field strength, advances in MR technology and analytics have enabled new applications, including detection of the microstructural environment, regional indices of dopaminergic and noradrenergic function, and changes in neurochemical metabolites. In fetal imaging, advances in image acquisition, such as multiband imaging or simultaneous multi slice approaches, which reduce acquisition times and enhance SNR, help address fetal motion and improve quantification of brain structure, diffusion, function, and metabolism during this critical neurodevelopmental period [13]. Fetal brain imaging offers the potential to identify altered and normative neurodevelopmental brain trajectories that likely impact future mental health with the hope of predicting risk in utero. Diffusion tensor imaging studies show compromised integrity of white matter microstructure in mental illnesses but without pathological specificity. In contrast, a recently developed multicompartment diffusion imaging model, neurite orientation dispersion and density imaging (NODDI), may provide better insight into gray and white matter microstructure [14], and the free-water diffusion imaging analysis method, which measures extracellular water, may provide insight changes in the extracellular matrix or neuroinflammation [15–17]. Another MR method with a recent application to psychiatry is neuromelanin-sensitive MRI. The output from the sequence is a contrast-to-noise ratio from the substantia nigra or locus coeruleus that is a proxy for dopaminergic or noradrenergic function. The output has been validated with post-mortem human data. Utilizing this technique may provide insight into the role of altered dopaminergic and noradrenergic function in different psychiatric conditions. Functional magnetic resonance spectroscopy is an evolving technique that assesses metabolite (e.g. glutamate, glutamine, lactate, and glucose) changes during a task [18]. This technique has potential to provide insight into altered neurotransmitter and metabolic dynamics in mental illness including how pharmacological agents functionally impact different brain regions.

## Multimodal approaches

As the capabilities of MR improve, the ability to acquire data beyond the second timescale and millimeter spatial resolution is evolving [19], and two examples are: the spatially resolved eddy current measurement (SPEEDI) sequence that can provide sub-millisecond temporal resolution [20] or the NexGen 7 T scanner with hardware advances that enables assessment of cortical layer functional activity [21]. Combination approaches such as fMRI + EEG, accompanied by increasingly sophisticated analytical techniques, can provide a unified perspective on the spatial and temporal pattern of whole-brain neural activity underlying behavioral states. Examples include multivariate analyses to understand how brain regions coordinate their activity using MR measurements and timed signals from electro- and magneto- encephalography (EEG and MEG) [22–24]. Another example is the combination of PET and MR imaging, which uses both a MR scanner and a PET scanner or a combined PET/MR system, that enables assessment of relationships between neurotransmitter systems with structural and functional abnormalities in various psychiatric disorders [25].

Another promising approach is to combine neuroimaging with brain stimulation. For example, transcranial magnetic stimulation (TMS) has been used to selectively manipulate brain subsystems that support cognitive domains of working memory, executive functions, and attention [26]. Future well-powered studies that combine brain stimulation approaches with fMRI, EEG, and computational modeling will be useful in bridging the gap in understanding how different brain states influence the effectiveness of brain stimulation on neurophysiological processes and behavior in healthy individuals and patient populations.

## Advances in animal neuroimaging

Concurrently with advances in human neuroimaging, there has been unprecedented progress in the domain of animal studies, particularly in rodents, using large-scale optical neural recordings. These in vivo approaches have enabled investigation of the coding and computational properties of large neuronal populations in behaving animals. For example, fluorescent calcium indicators of neural activity in combination with genetic and viral methods for their expression, chronic animal preparations for long-term/ high-speed imaging studies [27, 28], and devices to monitor and manipulate the activity of large neural ensembles, have provided unprecedented view of brain processing of information at the cellular level. Thus, these approaches have expanded our knowledge of brain coding in complex mental health-relevant behaviors. In recent years, in vivo measures using calcium indicators and causal neuronal manipulations (e.g. optogenetics) have been used concurrently with fMRI in rodents to relate more direct neural measures with resting state brain states derived noninvasively from BOLD signals [29, 30]. Advances have also been made with the use of genetically altered viruses to deliver opsins and indicators in nonhuman primates with recent studies providing evidence of GCaMP expression as applicable for two-photon Ca2+ imaging in macaques and marmosets during real time behavior [31–33]. Future studies with miniature microscopes and implanted microendoscopes may facilitate investigations of deeper cortical layers or subcortical regions in both rodents and nonhuman primates, paving the way to better understand brain function and how it becomes altered in mental illnesses.

## Data analysis & sharing

When combined with machine learning methods to decode and classify patterns of neural activity, neuroimaging outputs yield important insights into the temporal evolution of cognitive representations in the brain. These advances in imaging technology and analytics will accelerate the development of more accurate and predictive computational models of neural systems. Network modeling approaches, such as deep neural networks or hidden Markov models, can be used to simulate complex active and resting brain states [34], and this has led to novel theories of neural computations underlying cognitive processes in the human brain. A future challenge for computational modeling is to build more biologically accurate models that can simulate empirically-observed brain phenomena (e.g. oscillations and network states) and have predictive value for understanding brain function in health and psychopathology.

Greater access to neuroimaging data from NIH-supported studies (NOT-OD-21-013 and NOT-MH-23-100) has the potential to not only better inform existing computational models, but also test the validity of these models across a broader range of cognitive domains, transdiagnostic samples, or across the lifespan. Availability of high-value datasets such as Accelerating Medicines Partnership® Program for Schizophrenia (AMP-SCZ, https://www.ampscz.org) or the Adolescent Brain Cognitive Development^SM^ Study (https://abcdstudy.org/) encourages testing of new hypotheses.

To address issues of reproducibility [35, 36], NIMH requires the linkage of data shared in the NIMH Data Archive with published results in hopes that others can replicate study findings. NIMH has convened a National Advisory Mental Health Council workgroup on high dimensional datasets that is charged with generating recommendations regarding appropriate conceptual frameworks and experimental and analytical designs for studies using high dimensional datasets derived from neuroimaging data as well as other types of large datasets (e.g. “omics”) to promote rigor and reproducibility. The report from this workgroup is expected to be completed by the summer of 2024 and will be available on the NIMH website.

## Application of neuroimaging to advance treatment development

While considering the potential of novel neuroimaging approaches to transform the future of mental illness research, it is important to recognize that current methods are already playing important roles in research aimed at treatment development. For example, neuroimaging played a key role in the NIMH Fast-Fail approach, which attempts to develop biomarkers of target engagement and then Phase IIa proof of mechanism studies using changes in these biomarkers as the primary outcomes [37]. Using this approach, PET biomarkers of kappa opioid receptor (KOR) occupancy and fMRI measures of ventral striatal reward-related activity successfully demonstrated the potential of a kappa opioid antagonist for the treatment of anhedonia in mood and anxiety disorders. The Phase IIa trial demonstrated that engaging the KOR target pharmacologically achieved the predicted effect on ventral striatum activation during reward anticipation [38], and furthermore, that anhedonia was improved [39]. In addition, neuroimaging techniques are being utilized to identify neural signatures that predict risk for future psychiatric illness onset, clinical course trajectories, and biomarkers to guide treatment development. For instance, brain cortical thickness and resting-state functional connectivity are being investigated as potential biomarkers for predicting psychosis onset.

Emerging evidence supports the potential for neuroimaging to be key in personalizing therapies, particularly neuromodulatory interventions. For example, resting state fMRI has been used to define subtypes of depression (an approach called ‘biotyping’ where participants are grouped according to similar circuit dysfunction) which appear to be predictive of response to TMS [40]. Neuroimaging also features centrally in approaches to optimize and personalize the dosing of neuromodulation in space, time, and context [41]. Using structural neuroimaging to simulate the electric field induced in the brain by various forms of neuromodulation is an important part of understanding individual variation in clinical response to brain stimulation [42]. Using functional neuroimaging to identify person-specific targets for intervention has led to more effective and faster-acting TMS treatment protocols [43]. Indeed, person-specific neuroimaging is now part of the FDA labeling for the Magnus Neuromodulation System (MNS) with SAINT Technology for the treatment of depression. This system uses individualized fMRI targeting of the dorsolateral prefrontal cortex site that is most anticorrelated with subgenual anterior cingulate cortex [FDA 510(k) K220177]. As research yields more person-specific information regarding neurocircuitry underlying brain-based disorders, we may see future examples where neuroimaging becomes integral in an intervention targeting illness-specific circuitry.

## Summary and future directions

There are many new and exciting neuroimaging technologies emerging that have great potential to improve our understanding of the pathophysiology of mental illness. A challenge going forward is how to move these technologies from research into clinical practice. For instance, ongoing technological innovations to decrease the size of and increase the portability of human brain imaging systems can be expected to greatly accelerate research in the neural mechanisms of human social-cognitive interactions, as the recent pandemic has reminded us, is a significant factor for mental health. In addition, feasibility studies are needed to determine how to “scale-up” usage of these neuroimaging biomarkers in clinics and communities that may be under resourced. Finally, multi-disciplinary teams that combine clinical and technological expertise are needed to apply these emerging neuroimaging technologies to answer mental health-related questions and hopefully lead to translation to clinical care and improving patient lives and outcomes.
